# The association between diabetes mellitus and musculoskeletal disorders: a systematic review and meta-analysis

**DOI:** 10.3389/fendo.2024.1320468

**Published:** 2024-04-03

**Authors:** Mobin Azami, Asra Moradkhani, Maryam Afraie, Lotfolah Saed, Mohammad Amin Tapak, Kimya Khoramipoor, Sorour Khateri, Yousef Moradi

**Affiliations:** ^1^ Student of the Research Committee, Kurdistan University of Medical Sciences, Sanandaj, Iran; ^2^ Department of Epidemiology and Biostatistics, Faculty of Medicine, Kurdistan University of Medical Sciences, Sanandaj, Iran; ^3^ Department of Endocrinology, Faculty of Medicine, Kurdistan University of Medical Sciences, Sanandaj, Iran; ^4^ Department of Physical Medicine and Rehabilitation, School of Medicine, Sina (Farshchian) Educational and Medical Center, Hamadan University of Medical Sciences, Hamedan, Iran; ^5^ Social Determinants of the Health Research Center, Research Institute for Health Development, Kurdistan University of Medical Sciences, Sanandaj, Iran

**Keywords:** musculoskeletal disorders, diabetes mellitus, complications, evidence synthesis, meta-analysis

## Abstract

**Background:**

Despite the fact that DM patients are living longer, research on the prevalence of MSDs and other related illnesses is still lacking compared to that of other comorbidities. This study systematically reviewed and meta-analyzed cohort studies to determine the association between diabetes mellitus (DM) and musculoskeletal disorders (MSDs).

**Methods:**

A comprehensive search of international databases, including Medline (PubMed), Web of Science, Scopus, and Embase, was conducted up to June 2023 to identify relevant studies investigating the association between MSDs and DM.

**Results:**

The meta-analysis included ten cohort studies with a total of 308,445 participants. The pooled risk ratio (RR) estimate for the association between MSDs and DM was 1.03 (95% CI 1.00-1.06). Based on subgroup analysis, the association between longer duration (more than 7), European, below the age of 70, and female patients was higher than the others.

**Conclusion:**

In conclusion, the results of this meta-analysis suggest that there may be an association between MSDs and diabetes in people with diabetes. These findings add to the existing knowledge on this topic and highlight the importance of recognition and management of MSDs in people with DM. There is a need for further research to investigate the underlying mechanisms and to develop targeted interventions for the prevention and management of MSDs in this population.

**Systematic review registration:**

https://www.crd.york.ac.uk/prospero/display_record.php?RecordID=381787, identifier CRD42022381787.

## Introduction

Diabetes mellitus (DM) is a chronic disorder defined as persistent hyperglycemia (1). The prevalence of DM in 2011 was 366 million worldwide, which will rise to 552 million by 2030 (2). DM is associated with a range of complications, including both microvascular and macrovascular conditions. Microvascular complications of DM involve damage to the small blood vessels, particularly in the eyes (retinopathy) and nerves (neuropathy). Retinopathy can lead to vision impairment or even blindness, while neuropathy can result in numbness, tingling, or pain in the extremities. In severe cases, it may lead to foot ulcers or amputation. On the other hand, macrovascular complications of DM are related to large blood vessels and can affect various organs. Ischemic heart disease, in which blood flow to the heart muscles is reduced, and stroke, in which blood flow to the brain is interrupted, are two of the most common macrovascular problems that can happen because of DM. These conditions increase the risk of heart attacks and strokes in individuals with diabetes. Both microvascular and macrovascular complications contribute significantly to the morbidity and mortality associated with DM. Therefore, managing and preventing these complications are essential aspects of diabetes care and require comprehensive strategies targeting blood glucose control, blood pressure management, lipid control, and lifestyle modifications (3). Signs of MSDs associated with DM include muscle pain, joint pain or stiffness, reduced joint mobility, joint swelling, deformities, and a sensation of pins and needles in the arms or legs. Some MSDs are unique to individuals with diabetes. These complications significantly impact the quality of life and life expectancy of diabetic patients. Despite the increasing life expectancy of DM patients due to the availability of new antidiabetic drugs, the prevalence of MSDs and related disorders remains understudied compared to other complications (4–7).

The exact mechanism of MSDs in DM is unclear, but changes in collagen deposition and progressive non-enzymatic glycosylation in the connective tissue may be the cause (8, 9). MS complications affect different parts of the body. Soft tissue disorders such as cheiroarthropathy, carpal tunnel syndrome, trigger finger, Dupuytren’s contracture, and frozen shoulder can occur. Charcot arthropathy and gouty arthritis are examples of joint disease in people with diabetes. Bone involvement, such as osteoporotic and non-osteoporotic fractures and idiopathic skeletal hyperostosis, is seen (10, 11). Different places in patients with DM, such as the wrist, neck, spine, and knee, are involved (12). Diabetes duration, glucose level control, sex, and age are some risk factors for musculoskeletal complications (13, 14). Various studies, such as case-control or cohort studies, have been conducted around the world, but the results of these studies are controversial. This condition has implications for clinical and public health decision-making worldwide, particularly in developing countries. Determining the exact association between DM and MSDs may help clinicians and specialists reduce the impact and improve the quality of life of people with DM. As well as the results of this meta-analysis help develop and update clinical guidelines and improve evidence-based medicine (EBM) knowledge and policy in this field. Also, based on this information and the results of previous studies, DM is associated with musculoskeletal disorders (MSDs) and is often clinically underdiagnosed and undertreated. There have also been no systematic reviews of the literature to determine the association between DM and MSDs in the general population. Therefore, this study aimed to review systematically and meta-analyses the association between DM and MSDs by combining cohort studies.

## Materials and methods

This systematic review and meta-analysis were based on the Preferred Reporting Items for Systematic Reviews and Meta-analyses (PRISMA) (15). The study protocol was registered in PROSPER with the code CRD42022381787 (https://www.crd.york.ac.uk/prospero/display_record.php?RecordID=381787).

### Search terms and search strategy

A comprehensive search strategy was used to identify relevant trials for this meta-analysis. International databases, including Scopus, Web of Science, PubMed (Medline), and Embase, were searched using specific search terms and MeSH terms for ‘diabetes mellitus’ and ‘musculoskeletal disorders’. **Supplementary Table 1** provides detailed information on the systematic search. The search strategy for this meta-analysis covered the period up to July 2023. To ensure a comprehensive search, synonyms, and additional terms were included by reviewing other studies in the field. The first ten pages of Google Scholar were also searched, and related articles were selected. In addition, a manual search was conducted by reviewing the references to relevant articles. The collected articles were managed using Endnote software version 9. Duplicate articles were identified and removed based on the software’s default settings. Considering the predefined inclusion criteria, the remaining articles were evaluated based on their title, abstract, and full text. Two authors (MA and AM) independently and separately screened the articles based on their titles, abstracts, and full text. In cases of disagreement, the supervisor (YM) reviewed the results to reach a consensus.

### Inclusion and exclusion criteria

In this meta-analysis, we specifically included cohort studies that examined the association between DM and the occurrence of MSDs. The PECO structure was used to define the inclusion criteria:

Population: All patients with DM.
Exposure: Presence of MSDs.
Comparison: DM patients without MSDs.
Outcomes: Occurrence of MSDs and associated risk factors.

Cohort studies were selected for this meta-analysis because they can investigate causal associations in observational research. To maintain the focus on relevant studies, we excluded review articles, case reports, case series, clinical trials, other interventional studies, and letters to the editor. This exclusion criterion ensured that the included studies were cohort designs explicitly examining the relationship between DM and MSDs. By employing these inclusion and exclusion criteria, we aimed to conduct a meta-analysis using cohort studies to provide valuable insights into the association between DM and the occurrence of MSDs and their related risk factors.

### Data extraction

A checklist was developed to guide the data extraction process for this meta-analysis. The checklist included the first author’s name, country where the study was conducted, type of study (cohort study), study population, sample size, race/ethnicity of the study population, type of diabetes mellitus (DM), age of participants (mean and dispersion, if available), gender distribution (number of males), duration of DM, HbA1c levels, site(s) of involvement, type of MSDs, effect size (RR). Based on the checklist, two authors (MA and AM) independently performed the data extraction process. In cases where conflicts or disagreements arose, a third person (YM) was involved to resolve them and reach a consensus. This systematic data extraction approach collected the relevant information from each included study consistently and comprehensively, ensuring accuracy and minimizing bias in the subsequent analysis.

### Quality assessment

Two authors (MA and YM) assessed the quality of the included studies using the Joanna Briggs Institute (JBI) checklist for cohort and case-control studies. The JBI checklist is a validated tool that assesses the methodological quality of studies and the potential for bias in their design, conduct, and analysis. The JBI checklist consists of eleven cohort and ten case-control study questions. Each question is answered with ‘yes,’ ‘no,’ ‘not applicable,’ or ‘unclear’ to indicate whether the study adequately addressed the specific methodological criteria. Assessing the studies using the JBI checklist helped the authors determine the quality of the studies and decide whether to include or exclude them based on their quality assessment. It is worth noting that the JBI checklist has undergone extensive peer review and has been endorsed by the JBI Scientific Committee, further validating its usefulness for assessing the methodological quality of research studies (16, 17).

### Statistical analysis

The effect size in this meta-analysis was based on the risk ratio (RR) with a 95% confidence interval. Meta-set commands were used to assess the logarithm and log standard deviation of the RR. Cochrane I2 and Q tests were used to assess heterogeneity between trials. The Egger test was used to calculate publication bias. Subgroup analyses were performed based on the duration of DM, continent, fracture site, type of MSDs, age, and sex. Meta-regression analysis was also performed to determine the effect of age and BMI on the association of interest. Statistical analysis was performed using STATA 17.0, and a P value < 0.05 was considered.

## Results

### Study characteristics

**Figure 1:**
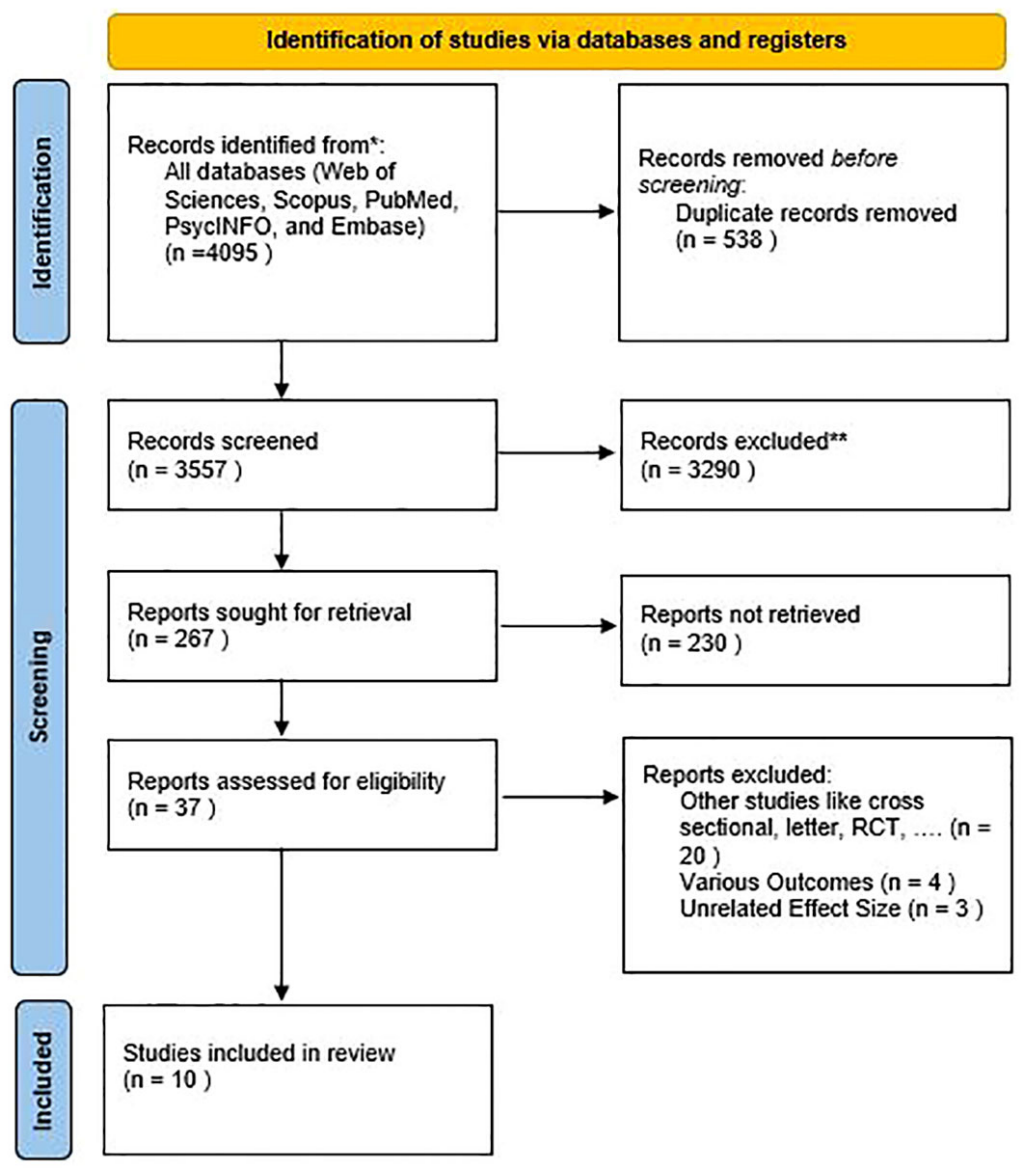
PRISMA 2020 flow diagram for new systematic reviews, which included searches of databases.

Based on the completion of the international database search, 4,095 studies were retrieved from PubMed, Scopus, Web of Science, and Embase databases. Following the removal of 538 duplicate studies, 3,557 studies remained. Subsequently, a screening process based on titles and abstracts resulted in 37 studies that met the eligibility criteria. Finally, after a thorough review of the full texts and consideration of inclusion and exclusion criteria, ten studies were included in our meta-analysis (**Figure 1**). The characteristics of the studies included in this meta-analysis have been reported in **Table 1**.

**Table 1:** The characteristics of included cohort studies.

Author (year)	Country	Type of study	Study population	Sample size	Age	Type of diabetes	Duration of DM	HbA1c	BMI	Place of involvement	Type of MS	Outcome
Strotmeyer et al. (2005) (18)	USA	Cohort	Older adults	2979	DM: 74 No DM: 73	Type 2 DM, IFG	DM and Fracture: 6.5	6.0 ± 0.5	NR	Radius/Ulna (21%)	Fracture	DM: 1.64 (1.07-2.51)
										Spine (18%)		
							DM and No Fracture: 6					
												IFG: 1.34 (0.67-2.67)
										Hip (18%)		
										Tibia/Fibula/Ankle (10%)		
										Foot (9%)		
Schwartz et al. (2001) (19)	USA	Cohort	Women aged 65 years and older	9654	72.0 ± 5.1	Type 2 Diabetes	9.2 ± 7.9	NR	28.8 ± 5.5	Hip (DM:48, No DM:501)	Fracture [Osteoarthritis:(DM:25, No DM:11)]	1.22 (1.06 -1.41)
										Knee (DM:24, No DM:258)		
										Foot (DM:23, No DM:264)		
Dobnig et al. (2006) (20)	Austria	Cohort	Female patients above 70 recruited in 95 nursing homes	1664	82.8 ± 5.9	Type 2 Diabetes	NR	6.5 ± 0.9	26.4 ± 4.6	Hip DM: 41 (7.0%) CTR: 69 (6.3%)	Fracture [Osteoarthritis:(DM:116, No DM:395]	0.90 (0.60 -1.34)
										Hand DM: 14 (2.4%) CTR: 21 (1.9%)		
										Other no vertebral fractures DM: 100 (9.2%) CTR: 48 (8.2%)		
Bonds et al. (2006) (21)	USA	Cohort	Postmenopausal women	93402	64.9 ± 7.0	Type 2 Diabetes	9.3 ± 10.0	NR	NR	Hip (DM:128, No DM:1531)	Fracture [Osteoarthritis:(DM:444, No DM:776]	1.20 (1.11–1.30)
										Hand (DM:117, No DM:3161)		
										Knee (DM:207, No DM:2828)		
										Foot (DM:153, No DM:1940)		
Ottenbacher et al. (2002) (22)	USA	Cohort	Mexican American Older Adults ≥ 65 years	2884	71.8 ± 5.7	NR	1213	NR	NR	Hip (DM:23, No DM:20)	Fracture	1.57(1.3-2.39)
Schwartz et al. (2011) (23)	USA	Cohort	–	16885	NR	Type 2 Diabetes	7436	NR	NR	Hip (DM:346, No DM:1275)	Fracture	Women/hip:1.88 (1.43-2.48)
												Women/non-spine:1.52 (1.31-1.75)
												Men/hip:5.71 (3.42-9.53)
												Men/non-spine:2.17 (1.75-2.69)
												Women/Osteoporotic:1.04 (1.02-1.05)
												Men/Osteoporotic:1.09 (1.04-1.14)
Napoli et al. (2014) (24)	USA	Cohort	men (≥65 years)	5994	73.3 ± 5.1	Type 2 Diabetes	5994	9.1 (2.7)	NR	Non-vertebral	Fracture	1.12 (0.94–1.34)
Martinez-Laguna et al. (2015) (25)	Spain	Cohort	NR	171931	62.71 ± 11.90	Type 2 Diabetes	97226	6 (1.69)	NR	Hip	Osteoporotic fractures T2DM: 444 Control:776	1.11 (0.99-1.24)
Guo et al. (2020) (26)	China	Cohort	Over 60 Years Old	3430	69:09 ± 6:53	Type 2 Diabetes	1229	NR	NR	–	Fracture (201) T2DM (78) Non-T2DM (123)	1.35 (1.00-1.82)
Sosa et al. (2009) (27)	Spain	Cohort	women aged more than 65 years old	203	71.7 ± 5.0	Type 2 Diabetes	All female	NR	NR	Hip (DM:1, No DM:0) Lumbar spine and femur	Fracture	1.04 (0.53-2.05)

### Association of DM with MSDs

**Figure 2:**
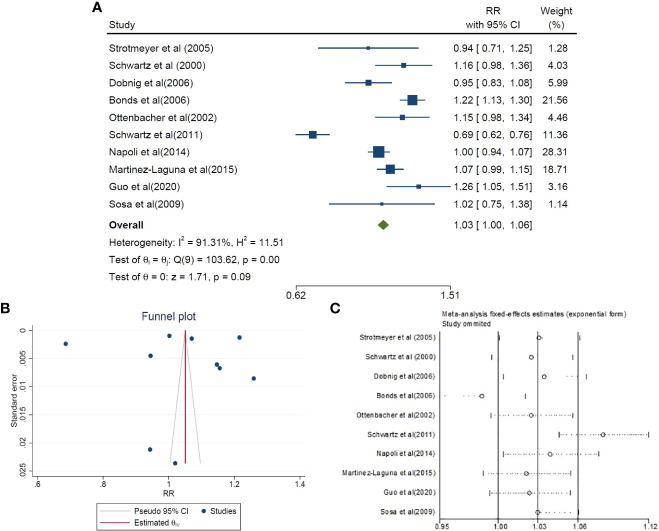
The risk ratio (RR) between DM and MSDs, (**A**: forest plot) sensitivity analysis **(C)** and publication bias **(B)** using a combination of the results of cohort studies (CI, Confidence Interval).

The present meta-analysis included 10 cohort studies comprising 69,979 patients with diabetes mellitus (DM) and 236,435 healthy controls. These studies aimed to assess the association between diabetes and MSDs. Gue et al. reported the highest RR of 1.26 (95% CI 1.05–1.51), while Schwartz et al. reported the lowest RR of 0.69 (95% CI 0.62–0.76). Upon combining these findings, the pooled RR estimate was 1.03 (95% CI 1.00–1.06), as depicted in **Figure 2**. The analysis revealed a substantial heterogeneity rate of 91.31 percent. Furthermore, an evaluation of publication bias in the included studies was conducted. The funnel plot showed significant publication bias across studies. However, the Egger's test results did not (B=-4.31; SE=3.645; P value=0.237), as shown in **Figure 2**.

### Subgroups analysis

Additionally, a subgroup analysis was performed in our study, considering various factors such as the duration of DM, continent, place of fracture, type of musculoskeletal disorder, age, and sex. The results of this subgroup analysis are summarized in **Table 2**.

**Table 2:** The subgroups analysis of association between DM and MSDs by combining cohort studies based on duration of DM, continents, age, and gender.

Variables	cat	No. study	Pooled RR (% 95 CI)	Heterogeneity Assessment between studies			Heterogeneity Assessment between subgroup	
				I2	P-value	Q	Q	P-value
Over all		10	1.03 (1.00 – 1.06)	91.31%	0.00	103.62	–	–
Duration of DM	<7	3	1.03 (0.97 – 1.10)	33.80%	0.22	3.02	2.12	0.15
	>7	3	1.09 (1.05 – 1.14)	88.48%	0.00	17.36		
Continent	America	6	1.02 (0.98 – 1.06)	94.78%	0.00	95.73	1.48	0.48
	Asia	2	1.04 (0.94 – 1.16)	84.17%	0.01	6.32		
	Europe	2	1.07 (0.99 – 1.15)	0.00%	0.76	0.09		
Age	<70	3	1.15 (1.10 – 1.21)	71.73%	0.03	6.99	12.57	0.00
	>70	6	1.02 (0.97– 1.07)	23.03%	0.26	6.50		
Sex	Female	4	1.15 (1.08 – 1.21)	74.19%	0.01	11.62	10.17	0.00
	male	1	1.00 (0.94 – 1.07)	–	0.00	0.00		

### Duration of DM

We divided the studies into two categories: more than seven years and less than seven years. Six trials were assessed, and each group contained three trials. In the more than seven-years group, the pooled estimate of RR was 1.09 (95% CI 1.05-1.14), but in the less than seven-years group, it was 1.03 (95% CI 0.97-1.10; P-value=0.00, 0.22) with heterogeneity (I2) of 88.48% and 33.80%, respectively (**Table 2**).

### Continent

Studies have been conducted on three continents: six in America, two in Asia, and two in Europe. Our findings revealed that the combined RR estimate was 1.02 (95% CI 0.98–1.06; P-value=0.00) in America, 1.04 (95% CI 0.94–1.16; P-value=0.01) in Asia, and 1.07 (95% CI 0.99–1.15; P-value=0.76) in Europe, accompanied by heterogeneity (I2) rates of 94.78%, 84.17%, and 0.00% respectively. Notably, the risk of MSDs in patients with diabetes mellitus (DM) was found to be higher in Europe compared to America. At the same time, no significant association was observed in Asia (**Table 2**).

### Age and gender

We categorized the studies based on age, specifically comparing those below 70 with those above it. Three articles were analyzed for the lower age group, revealing a pooled RR estimate of 1.15 (95% CI 1.10–1.21; P-value=0.03, I2 = 71.73%). However, in the higher age group comprising six articles, the pooled RR estimate was 1.02 (95% CI 0.97–1.07; P-value=0.26, I2 = 23.03%). These findings indicate that the risk of MSDs is higher in individuals below the age of 70 (**Table 2**).

Furthermore, we observed differences based on sex. Four studies were included in the analysis of the pooled RR estimate for females, resulting in a value of 1.15 (95% CI 1.08–1.21; P-value=0.01). Conversely, only one study evaluated the RR for males, which was 1.00 (95% CI 0.94–1.07) (**Table 2**).

### Place of the fracture

Among the articles that examined fractures, they were categorized into four groups based on the specific location. Six of these articles specifically investigated hip, pelvis, and upper leg fractures, resulting in a pooled RR estimate of 1.53 (95% CI 1.42–1.65). No significant differences were observed in other locations (**Table 3**). In six of the articles, the focus was specifically on hip disorders. The study by Dobing et al. reported the lowest RR of 1.07 (95% CI 0.79-1.45), whereas the study by Schwartz et al. reported the highest RR of 2.01 (95% CI 1.79-2.25). The pooled RR estimate for hip involvement was 1.53 (95% CI 1.42–1.65; P-value=0.00, I2 = 88.73%) (**Table 3**). In addition to these results, 2 studies reported the outcomes of interest based on the inclusion criteria for arm/wrist/hand fracture and 2 studies also reported lower leg/ankle/knee fracture. After combining these results, the meta-analysis showed that the association between the presence of diabetes and arm/wrist/hand fracture was 0.96 (% 95 CI 0.83 - 1.10) and for lower leg/ankle/knee fracture was 1.22 (% 95 CI 1.07 - 1.38). Similarly, the association between diabetes and foot fracture was 1.28 (% 95 CI 1.11 - 1.48) (**Table 3**).

**Table 3:** The subgroups analysis of association between DM and MSDs by combining cohort studies based on place of fracture, type of MSDs.

Variables	cat	No. study	Pooled RR (% 95 CI)	Heterogeneity Assessment between studies			Heterogeneity Assessment between subgroup	
				I2	P-value	Q	Q	P-value
Fracture	hip/pelvis/upper leg	6	1.53 (1.42 – 1.65)	88.73%	0.00	44.38	11.14	0.00
	lower arm/wrist/hand	2	0.96 (0.83 – 1.10)	0.00%	0.36	0.83	-0.64	0.52
	lower leg/ankle/knee	2	1.22 (1.07 – 1.38)	0.00%	0.86	0.03	3.05	0.0
	foot	2	1.28 (1.11 – 1.48)	0.00%	0.67	0.19	3.40	0.00
Type of MSDs	Osteoarthritis	3	4.88 (4.55 – 5.22)	99.72%	0.00	709.16	45.17	0.00

### Types of MSDs

Osteoarthritis was among the disorders examined in several studies. The combined RR estimate for the three studies was 4.88 (95% CI 4.55–5.22; P-value=0.00, I2 = 99.72%). This finding indicates that DM could be a significant risk factor for osteoarthritis (**Table 3**).

### Meta-regression results

This analysis examined the relationship between our results and three variables: age, BMI, and year. The regression coefficients and statistical significance for each variable were as follows: age (B: -0.009, SE: 0.005, P: 0.096), BMI (B: -0.007, SE: 0.022, P: 0.746), and year (B: -0.001, SE: 0.01, P: 0.890). The age variable demonstrated a weakly decreasing relationship but did not reach statistical significance. The variables of BMI and year did not show any significant associations with our results.

### Quality assessment

The quality of 10 cohort studies was evaluated using the Joanna Briggs Institute (JBI) critical appraisal tools. The assessment revealed that the majority of the cohort studies received high-quality scores, indicating a high level of methodological rigor (**Table 4**).

**Table 4:** Quality assessment of cohort studies based on the JBI critical appraisal checklist.

Studies	Q1	Q2	Q3	Q4	Q5	Q6	Q7	Q8	Q9	Q10	Q11	Total Score
Strotmeyer et al. (2005) (18)	Y	Y	Y	Y	Y	Y	Y	N	Y	UC	Y	9
Schwartz et al. (2001) (19)	Y	Y	Y	Y	N	Y	Y	Y	Y	Y	Y	10
Dobnig et al. (2006) (20)	Y	Y	Y	Y	Y	Y	Y	Y	Y	N	Y	10
Bonds et al. (2006) (21)	Y	Y	Y	Y	Y	Y	Y	Y	Y	Y	Y	11
Ottenbacher et al. (2002) (22)	Y	Y	Y	Y	Y	Y	Y	Y	Y	Y	Y	11
Schwartz et al. (2011) (23)	Y	Y	Y	Y	Y	Y	Y	Y	N	N	Y	9
Napoli et al. (2014) (24)	Y	Y	Y	Y	Y	Y	Y	Y	Y	Y	Y	11
Martinez-Laguna et al. (2015) (25)	Y	Y	Y	Y	Y	Y	Y	Y	Y	N	Y	10
Guo et al. (2020) (26)	Y	Y	Y	Y	Y	Y	Y	N	UC	Y	Y	9
Sosa et al. (2009) (27)	Y	Y	Y	Y	Y	Y	Y	N	Y	Y	Y	10

Q1: Were the two groups similar and recruited from the same population?

Q2: Were the exposures measured similarly to assign people to both exposed and unexposed groups?

Q3: Was the exposure measured in a valid and reliable way?

Q4: Were confounding factors identified?

Q5: Were strategies to deal with confounding factors stated?

Q6: Were the groups/participants free of the outcome at the start of the study (or at the moment of exposure)?

Q7: Were the outcomes measured in a valid and reliable way?

Q8: Was the follow up time reported and sufficient to be long enough for outcomes to occur?

Q9: Was follow up complete, and if not, were the reasons to loss to follow up described and explored?

Q10: Were strategies to address incomplete follow up utilized?

Q11: Was appropriate statistical analysis used?

Y, YES; N, NO; UC, UNCLEAR; NP, Not applicable.

## Discussion

This meta-analysis aimed to evaluate the association between DM and MSDs. Various categories were considered in the analysis, including the duration of DM, continent, place of fracture, type of MSDs, age, and sex. The results consistently indicated that individuals with DM have a higher risk of developing musculoskeletal complications than the general population. These findings align with previous studies and further support the association between DM and MSDs. Indeed, studies have demonstrated that DM affects the human body in various ways, although the exact mechanisms are not fully understood. One proposed mechanism involves increased levels of advanced glycation end-products (AGEs), which can trigger fibroproliferative complications. When the receptor for AGEs (RAGE) is turned on, it activates several signaling pathways, such as the MAPK/ERK, TGF-β, JNK, and NF-κB pathways (28). This activation leads to two main complications. Firstly, it induces oxidative stress in the endoplasmic reticulum (ER) (29). Secondly, it upregulates the expression of inflammatory cytokines such as IL-1β, IL-6, and TNFα (28, 30–32). These pathways can influence proteins like collagen, making them more susceptible to glycation (33). Histologic studies have revealed that this mechanism affects individuals with diabetes, as changes in AGE pathways can lead to decreased elasticity and load-bearing capacity of tendons (34, 35). Additionally, chondrocytes, the cells responsible for cartilage maintenance, can adapt their expression of glucose transporters (GLUT-1, GLUT-3, and GLUT-9) in response to blood glucose levels. However, this adaptive mechanism may be absent in MSDs such as osteoarthritis. TNFα and IL-1β can make IL-6, PGE2, and phosphokinase C work, which controls the amount of expressed GLUT transporters (36–38). Therefore, hyperglycemia and insulin resistance in diabetes can alter these pathways and impact the musculoskeletal system.

In the subgroup analysis, the association between DM and MSDs was found to vary across different categories. Specifically, diabetic patients with a longer duration of DM were observed to have a higher risk of developing musculoskeletal complications. This finding can be attributed to the cumulative effects of prolonged exposure to diabetes, which can lead to both microvascular and macrovascular complications. Over time, diabetes can adversely affect the small blood vessels (microvascular) and larger blood vessels (macrovascular) throughout the body. These vascular complications can impair blood flow, nutrient delivery, and oxygen supply to the musculoskeletal system, increasing the risk of musculoskeletal problems. It is important to note that the longer the duration of DM, the higher the likelihood of developing microvascular complications such as diabetic neuropathy, retinopathy, and nephropathy. These complications can contribute to the development of musculoskeletal issues by affecting nerve function, blood supply, and overall tissue health. As a result, the subgroup analysis shows that the length of DM is strongly related to the risk of musculoskeletal complications. The longer the DM, the higher the risk, as the microvascular and macrovascular complications increase over time (39). Our results were confirmed in the study of Bonds et al. in 2006 (21). Indeed, age and sex differences have been identified as potential risk factors for MSDs in diabetic patients. Their study found musculoskeletal complications were more prevalent in younger and female individuals. This observation aligns with the existing literature and can be explored from different perspectives, including the influence of sex hormones. Sex hormones, such as estrogen, play a role in regulating the health and function of tendons and ligaments. Estrogen has been shown to have a protective effect on these connective tissues, promoting their strength and elasticity. In females, the decline in estrogen levels during menopause can contribute to changes in tendon and ligament properties, making them more susceptible to injury or degeneration. Moreover, hormonal differences between males and females can also impact the inflammatory response and immune function, which may contribute to the development and progression of MSDs. Additionally, differences in body composition, muscle mass, and biomechanics between sexes may influence the distribution of forces and loading patterns on the musculoskeletal system, potentially increasing the risk of certain conditions. Considering age, it is essential to note that younger individuals may be more susceptible to musculoskeletal complications due to longer exposure to diabetes-related metabolic abnormalities and prolonged disease duration. Furthermore, age-related factors such as decreased tissue regeneration capacity, increased oxidative stress and cumulative damage over time can contribute to a higher risk of MSDs in older individuals. By considering these age and sex-related factors, a more comprehensive understanding of the underlying mechanisms and risk factors associated with MSDs in diabetic patients can be achieved (40). Second is diversity in muscle fibers; for example, women have more type I fibers than others (41). Third, lower pain thresholds in females can be the other cause (42). One of the specific diseases investigated in the diabetic population was osteoarthritis, and our study revealed a heightened risk of its occurrence. This increased risk can be attributed to elevated levels of pro-inflammatory cytokines, oxidative stress, obesity, and higher secretion of adipokines in individuals with diabetes. These factors play significant roles in the development and progression of osteoarthritis (43, 44). Our findings approved Courties et al.’s previous study 2016 (45).

Future research should focus on identifying additional factors contributing to the increased risk of MSDs in diabetic individuals, such as specific treatment modalities, comorbidities, lifestyle factors, or genetic predisposition. By expanding our knowledge of the risk factors and underlying mechanisms, healthcare providers can develop more targeted strategies for prevention, early detection, and effective management of musculoskeletal complications in individuals with diabetes. This will ultimately improve patient outcomes and contribute to the development of evidence-based guidelines for clinical practice.

The study possesses several strengths that contribute to its significance. Firstly, it stands as the first systematic review and meta-analysis to investigate and analyze the association between DM and MSDs comprehensively. This approach enhances the overall understanding of the topic and provides a valuable synthesis of existing evidence. Secondly, the study employed diverse studies that investigated various outcomes related to MSDs. This allowed for a subgroup analysis based on different specifications, enabling a more nuanced association examination. The high homogeneity observed in the results indicates that the included articles were appropriately chosen, further strengthening the validity of the findings. Thirdly, using cohort studies for meta-analysis provides a foundation for exploring causal associations between DM and MSDs. This approach allows for incorporating genetic and molecular science studies, which can shed light on underlying mechanisms and potential pathways. However, it is important to acknowledge certain limitations of the study. One such limitation is the lack of analysis based on confounding variables. MSDs can develop and advance as a result of factors like the type of treatment diabetic patients receive and the presence of other underlying diseases. The absence of consideration for these confounders restricts the ability to explain the study results fully and may leave room for alternative interpretations. Despite these limitations, the study’s strengths, including its systematic and comprehensive approach, subgroup analysis, and utilization of cohort studies, contribute to its valuable insights into the association between diabetes and musculoskeletal disorders. Despite study limitations, further research is crucial to understand the underlying mechanisms, potentially informing targeted interventions that can mitigate MSDs prevalence and impact in the diabetic population. This underscores the importance of an integrated healthcare approach, ultimately enhancing the overall quality of life for affected individuals.

## Conclusion

In conclusion, our findings shed light on an understudied aspect of diabetes-related comorbidities, emphasizing the nuanced nature of this relationship, particularly in specific subgroups. Importantly, they highlight the need for a holistic approach to diabetes management, addressing both glycemic control and musculoskeletal health.

## Data availability statement

The original contributions presented in the study are included in the article/**Supplementary Material**. Further inquiries can be directed to the corresponding authors.

## Author contributions

MAz: Conceptualization, Investigation, Methodology, Project administration, Supervision, Writing – original draft, Writing – review & editing. AM: Data curation, Writing – original draft. MAf: Formal analysis, Methodology, Writing – original draft. LS: Data curation, Investigation, Writing – original draft. MT: Data curation, Writing – original draft. KK: Data curation, Investigation, Writing – original draft. SK: Conceptualization, Data curation, Investigation, Project administration, Writing – original draft, Writing – review & editing. YM: Conceptualization, Data curation, Investigation, Methodology, Software, Supervision, Writing – review & editing.
