# Supplementary material for: The association between diabetes mellitus and musculoskeletal disorders: a systematic review and meta-analysis
Source: Front Endocrinol (Lausanne). 2024 Apr 3;15:1320468. doi: 10.3389/fendo.2024.1320468 (PMC11022848; doi:10.3389/fendo.2024.1320468)
Supplement: Supplementary file 1 [file Table_1.docx]

| Databases | Search Syntax | No. |
| --- | --- | --- |
| PubMed | ("Diabetes Mellitus"[Title/Abstract] OR "Insulin-Dependent Diabetes Mellitus"[Title/Abstract] OR "Juvenile-Onset Diabetes Mellitus"[Title/Abstract] OR "IDDM"[Title/Abstract] OR "juvenile onset diabetes"[Title/Abstract] OR "juvenile onset diabetes"[Title/Abstract] OR "Sudden-Onset Diabetes Mellitus"[Title/Abstract] OR "Type 1 Diabetes Mellitus"[Title/Abstract] OR "insulin dependent diabetes mellitus 1"[Title/Abstract] OR "insulin dependent diabetes mellitus 1"[Title/Abstract] OR "Type 1 Diabetes"[Title/Abstract] OR "Autoimmune Diabetes"[Title/Abstract] OR "Brittle Diabetes Mellitus"[Title/Abstract] OR "Ketosis-Prone Diabetes Mellitus"[Title/Abstract] OR "Ketosis-Resistant Diabetes Mellitus"[Title/Abstract] OR "Non-Insulin-Dependent Diabetes Mellitus"[Title/Abstract] OR "Stable Diabetes Mellitus"[Title/Abstract] OR "NIDDM"[Title/Abstract] OR "maturity onset diabetes mellitus"[Title/Abstract] OR "maturity onset diabetes mellitus"[Title/Abstract] OR "MODY"[Title/Abstract] OR "Type 2 Diabetes Mellitus"[Title/Abstract] OR "noninsulin dependent diabetes mellitus"[Title/Abstract] OR "noninsulin dependent diabetes mellitus"[Title/Abstract] OR "maturity onset diabetes"[Title/Abstract] OR "maturity onset diabetes"[Title/Abstract] OR "Type 2 Diabetes"[Title/Abstract] OR "Adult-Onset Diabetes Mellitus"[Title/Abstract] OR "Pregnancy-Induced Diabetes"[Title/Abstract] OR "Gestational Diabetes Mellitus"[Title/Abstract] OR "Gestational Diabetes"[Title/Abstract] OR "Prediabetic States"[Title/Abstract] OR "Prediabetes"[Title/Abstract]) AND ("Musculoskeletal Diseases"[Title/Abstract] OR "Musculoskeletal Disease"[Title/Abstract] OR "Orthopedic Disorders"[Title/Abstract] OR "Orthopedic Disorder"[Title/Abstract] OR "Bone Diseases"[Title/Abstract] OR "Bone Disease"[Title/Abstract] OR "Cartilage Diseases"[Title/Abstract] OR "Cartilage Disease"[Title/Abstract] OR "Foot Disease"[Title/Abstract] OR "Foot Diseases"[Title/Abstract] OR "Joint Disease"[Title/Abstract] OR "Joint Diseases"[Title/Abstract] OR "Muscular Diseases"[Title/Abstract] OR "Muscular Disease"[Title/Abstract] OR "Muscle Disorders"[Title/Abstract] OR "Muscle Disorder"[Title/Abstract] OR "Musculoskeletal Abnormalities"[Title/Abstract] OR "Musculoskeletal Abnormality"[Title/Abstract]) | 882 |
| Scopus | ( ( TITLE-ABS-KEY ( "Diabetes Mellitus" ) OR TITLE-ABS-KEY ( "Insulin-Dependent Diabetes Mellitus" ) OR TITLE-ABS-KEY ( "Juvenile-Onset Diabetes Mellitus" ) OR TITLE-ABS-KEY ( iddm ) OR TITLE-ABS-KEY ( "juvenile onset diabetes" ) OR TITLE-ABS-KEY ( "juvenile onset diabetes" ) OR TITLE-ABS-KEY ( "Type 1 Diabetes Mellitus" ) OR TITLE-ABS-KEY ( "insulin dependent diabetes mellitus 1" ) OR TITLE-ABS-KEY ( "insulin dependent diabetes mellitus 1" ) OR TITLE-ABS-KEY ( "Type 1 Diabetes" ) OR TITLE-ABS-KEY ( "Autoimmune Diabetes" ) OR TITLE-ABS-KEY ( "Non-Insulin-Dependent Diabetes Mellitus" ) OR TITLE-ABS-KEY ( "Stable Diabetes Mellitus" ) OR TITLE-ABS-KEY ( niddm ) OR TITLE-ABS-KEY ( "maturity onset diabetes mellitus" ) OR TITLE-ABS-KEY ( "maturity onset diabetes mellitus" ) OR TITLE-ABS-KEY ( "Type 2 Diabetes Mellitus" ) OR TITLE-ABS-KEY ( "noninsulin dependent diabetes mellitus" ) OR TITLE-ABS-KEY ( "noninsulin dependent diabetes mellitus" ) OR TITLE-ABS-KEY ( "Type 2 Diabetes" ) OR TITLE-ABS-KEY ( "Adult-Onset Diabetes Mellitus" ) OR TITLE-ABS-KEY ( "Pregnancy-Induced Diabetes" ) OR TITLE-ABS-KEY ( "Gestational Diabetes Mellitus" ) OR TITLE-ABS-KEY ( "Gestational Diabetes" ) OR TITLE-ABS-KEY ( "Prediabetic States" ) OR TITLE-ABS-KEY ( "Prediabetes" ) ) ) AND ( ( TITLE-ABS-KEY ( "Musculoskeletal Diseases" ) OR TITLE-ABS-KEY ( "Musculoskeletal Disease" ) OR TITLE-ABS-KEY ( "Orthopedic Disorders" ) OR TITLE-ABS-KEY ( "Orthopedic Disorder" ) OR TITLE-ABS-KEY ( "Musculoskeletal Abnormalities" ) OR TITLE-ABS-KEY ( "Musculoskeletal Abnormality" ) ) ) | 2377 |
| Web of science | ((TS=("Diabetes Mellitus") OR TS=("Insulin-Dependent Diabetes Mellitus") OR TS=("Juvenile-Onset Diabetes Mellitus") OR TS=("IDDM") OR TS=("juvenile onset diabetes") OR TS=("juvenile onset diabetes") OR TS=("Type 1 Diabetes Mellitus") OR TS=("insulin dependent diabetes mellitus 1") OR TS=("Type 1 Diabetes") OR TS=("Autoimmune Diabetes") OR TS=("Non-Insulin-Dependent Diabetes Mellitus") OR TS=("Stable Diabetes Mellitus") OR TS=("NIDDM") OR TS=("maturity onset diabetes mellitus") OR TS=("maturity onset diabetes mellitus") OR TS=("MODY") OR TS=("Type 2 Diabetes Mellitus") OR TS=("noninsulin dependent diabetes mellitus") OR TS=("noninsulin dependent diabetes mellitus") AND TS=("maturity onset diabetes") OR TS=("maturity onset diabetes") OR TS=("Type 2 Diabetes") OR TS=("Pregnancy-Induced Diabetes") OR TS=("Gestational Diabetes Mellitus") OR TS=("Gestational Diabetes") OR TS=("Prediabetic States") OR TS=("Prediabetes")) AND (TS=("Musculoskeletal Diseases") OR TS=("Musculoskeletal Disease") OR TS=("Orthopedic Disorders") OR TS=("Orthopedic Disorder") OR TS=("Bone Diseases") OR TS=("Bone Disease") OR TS=("Cartilage Diseases") OR TS=("Cartilage Disease") OR TS=("Foot Disease") OR TS=("Foot Diseases") OR TS=("Joint Disease") OR TS=("Joint Diseases") OR TS=("Muscular Diseases") OR TS=("Muscular Disease") OR TS=("Muscle Disorders") OR TS=("Muscle Disorder") OR TS=("Musculoskeletal Abnormalities") OR TS=("Musculoskeletal Abnormality"))) | 836 |
